# Evolutionary Diversification of Primary Metabolism and Its Contribution to Plant Chemical Diversity

**DOI:** 10.3389/fpls.2019.00881

**Published:** 2019-07-10

**Authors:** Hiroshi A. Maeda

**Affiliations:** Department of Botany, University of Wisconsin–Madison, Madison, WI, United States

**Keywords:** plant chemical diversity, metabolic enzymes, primary metabolism, specialized metabolism, evolution of plant metabolism, amino acid biosynthesis

## Abstract

Plants produce a diverse array of lineage-specific specialized (secondary) metabolites, which are synthesized from primary metabolites. Plant specialized metabolites play crucial roles in plant adaptation as well as in human nutrition and medicine. Unlike well-documented diversification of plant specialized metabolic enzymes, primary metabolism that provides essential compounds for cellular homeostasis is under strong selection pressure and generally assumed to be conserved across the plant kingdom. Yet, some alterations in primary metabolic pathways have been reported in plants. The biosynthetic pathways of certain amino acids and lipids have been altered in specific plant lineages. Also, two alternative pathways exist in plants for synthesizing primary precursors of the two major classes of plant specialized metabolites, terpenoids and phenylpropanoids. Such primary metabolic diversities likely underlie major evolutionary changes in plant metabolism and chemical diversity by acting as enabling or associated traits for the evolution of specialized metabolic pathways.

## Introduction

Plants produce a diverse array of secondary or specialized metabolites, which play critical roles in plant adaptation under various environmental conditions. These phytochemicals are also widely used in human nutrition and medicine. Nearly one million metabolites are estimated to be produced throughout the plant kingdom (Afendi et al., 2012), though many of them are yet to be discovered. All of these specialized metabolites are synthesized from a certain primary metabolite precursor(s), such as sugars, amino acids, nucleotides, organic acids, and fatty acids, which are essential for maintaining cellular homeostasis and the life of whole organisms. Besides their vital nature, primary metabolic pathways are highly regulated and integrated to complex metabolic networks (Baghalian et al., 2014; Sulpice and McKeown, 2015; Beckers et al., 2016; Filho et al., 2018). Consequently, genes encoding primary metabolic enzymes are subjected to purifying selection and generally considered to be conserved among the plant kingdom, unlike highly diversifiedspecialized metabolism (Pichersky and Lewinsohn, 2011; Weng et al., 2012; Moghe and Last, 2015; Moore et al., 2019). Yet, some primary metabolic pathways were altered during plant evolution, which had profound impacts on overall plant physiology, metabolism, and adaptation. This review describes examples of primary metabolic diversification in different plant lineages and discusses their potential roles in the evolution of downstream specialized metabolic pathways and plant chemical diversity as enabling or associated traits.

## Enablers of Evolutionary Diversification of the Photosynthetic Carbon Fixation Pathways

**FIGURE 1:**
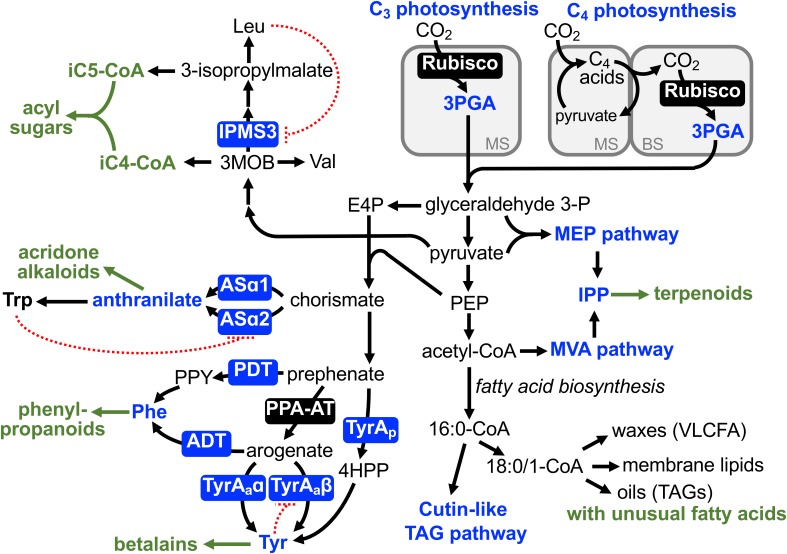
Diversification of primary metabolic pathways and enzymes in plants. Although primary metabolism is highly constrained and generally assumed to be conserved within the plant kingdom, there are examples of evolutionary diversification of some primary metabolic pathways (blue). Many of them likely supported diversification of downstream specialized metabolism (green letters) as enabling or associated traits. Blue letters and boxes denote alternative routes or enzymes to synthesize key primary metabolite precursors. Green arrows and letters indicate specialized metabolic pathways derived from these primary precursors. Dotted red lines indicate feedback inhibition that act specifically on canonical leucine (Leu), tryptophan (Trp), and tyrosine (Tyr) biosynthetic pathways, but not on “lineage-specific” alternative enzymes. ADT, arogenate dehydratase; ASα, anthranilate synthase α subunit; BS, bundle sheath cells; E4P, erythrose 4-phosphate; 4HPP, 4-hydroxyphenylpyruvate; iC4-CoA, 2-methylpropanoic-coenzyme A; iC5-CoA, 2-methylbutanoic-coenzyme A; IPMS, isopropylmalate synthase; IPP, isopentenyl diphosphate; MEP, methylerythritol phosphate; 3MOB, 3-methyl-2-oxobutanoate; MS, mesophyll cells; MVA, mevalonate; PDT, prephenate dehydratase; PEP, phospho*enol*pyruvate; 3PGA, 3-phosphoglycerate; PPA-AT, prephenate aminotransferase; PPY, phenylpyruvate; TAG, triacylglycerol; TyrAa, arogenate dehydrogenase; TyrAp, prephenate dehydrogenase; Val, valine; VLCFA, very long chain fatty acid.

One of the most fundamental metabolic pathways of plants, photosynthetic carbon fixation, has been modified in a number of plant lineages to what is known as C_4_ photosynthesis and Crassulacean acid metabolism, though the former will be mainly discussed here. Unlike 3-phosphoglycerate (3PGA), a three carbon molecule produced by ribulose-1,5-bisphosphate carboxylase/oxygenase (Rubisco) in C_3_ photosynthesis, C_4_ photosynthesis initially generates a four carbon molecule, i.e., oxaloacetate, by phospho*enol*pyruvate (PEP) carboxylase (PEPC). Oxaloacetate is further converted to malate or aspartate and shuttled from mesophyll to bundle sheath cells, where CO_2_ is released for refixation by Rubisco (Figure 1) (Langdale, 2011; Sage et al., 2012; Furbank, 2016). This highly intricate mechanism is seemingly maladaptive due to high metabolic costs (e.g., fixing carbon twice, regeneration of PEP), but provides adaptive advantage under arid, warm, and high light conditions by concentrating CO_2_ and attenuating the oxygenation side reaction of Rubisco and hence photorespiration (Christin and Osborne, 2014; Sage and Stata, 2015). Thus, besides the decline in atmospheric CO_2_ around 30 million years ago (Pagani et al., 2005), such extreme environmental conditions, in which some plants existed, likely acted as an “environmental enabler” for the evolutionary diversification of the photosynthetic carbon fixation, the entry step of plant metabolic pathways.

The C_4_ photosynthetic pathway evolved more than 60 times independently across the plant phylogeny (Sage et al., 2011, 2012). Notably, C_4_ photosynthesis is unevenly distributed across the phylogeny and particularly prevalent in specific plant lineages, such as Poaceae and Caryophyllales (Christin et al., 2009, 2015; Sage et al., 2011). Recent comparative analyses of C_3_ and C_4_ plants as well as C_3_-C_4_ transitory species revealed that the repeated evolution of C_4_ photosynthesis was likely facilitated by certain “pre-conditions” or “enabling traits” that emerged or were present in certain plant lineages (Ludwig, 2013; Sage et al., 2014; Heckmann, 2016; Miyake, 2016; Schlüter and Weber, 2016). These enabling traits include “genetic enablers,” such as C_4_-like cell-type specific expression of C_4_ enzymes (e.g., PEPC, Williams et al., 2012; Christin et al., 2013a, 2015) and “anatomical enablers,” such as proto-Kranz anatomy (Christin et al., 2013b; Lundgren et al., 2014; Sage et al., 2014), in C_3_ ancestors. These pre-conditions further facilitated emergence of “metabolic enablers,” such as shuttling of photorespiratory glycine from mesophyll to bundle sheath cells acting as CO_2_ pump (Sage et al., 2013; Schulze et al., 2013). This so-called C_2_ photosynthesis is present in many sister species to C_4_ lineages (Sage et al., 2011, 2012; Khoshravesh et al., 2016) and appears to be accompanied by shuttling of other metabolites, such as alanine/pyruvate or aspartate/malate, for balancing of nitrogen between the mesophyll and bundle sheath cells (Mallmann et al., 2014; Schlüter and Weber, 2016). Once these pre-conditions were established, C_4_ photosynthesis could evolve relatively easily and thus repeatedly, such as through optimization of kinetic properties of C_4_ enzymes (e.g., PEPC) and bundle sheath specific expression of Rubisco (Langdale, 2011; Sage et al., 2012; Furbank, 2016; Reeves et al., 2017). Thus, the combination of environmental, genetic, anatomical, and metabolic enablers allowed astounding alterations in the core primary metabolic pathway, photosynthetic carbon fixation, in certain plant lineages.

## Diversification of Amino Acid Biosynthetic Pathways at the Interface of Primary and Specialized Metabolic Pathways

Amino acid biosynthetic pathways not only provide essential protein building blocks but connect central carbon metabolism to a variety of specialized metabolism. Some of these amino acid pathways have diversified in certain plant lineages and likely contributed to the chemical diversity of their downstream specialized metabolism.

Isopropylmalate synthase (IPMS) catalyzes the committed step of leucine biosynthesis (de Kraker et al., 2007). IPMS competes for the 3-methyl-2-oxobutanoate (3MOB) substrate with valine biosynthesis (Figure 1) and is typically feedback inhibited by the end product, leucine, through its C-terminal allosteric regulatory domain (Koon et al., 2004; de Kraker and Gershenzon, 2011). Glandular trichomes of Solanaceae plants accumulate insecticidal specialized metabolites, acylsugars, which have various aliphatic acids attached to a sugar backbone (e.g., sucrose, Fan et al., 2019). A wild tomato *Solanum pennellii* and the cultivated tomato, *Solanum lycopersicum*, have 2-methylpropanoic and 3-methylbutanoic acid (iC4 and iC5) acyl chains, which are derived from 3MOB and 3-isopropylmalate, intermediates of valine and leucine metabolism, respectively (Figure 1). Analysis of introgression lines between *S. lycopersicum* and *S. pennellii*, followed by expression and biochemical analyses, revealed that the C-terminal regulatory domain of the IPMS3 isoform is truncated in *S. lycopersicum*, making this isoform insensitive to leucine-mediated feedback inhibition (Schilmiller et al., 2010; Ning et al., 2015). In contrast, the IPMS3 isoform of *S. pennellii* is further truncated into its catalytic domain and has lost the enzyme activity. Thus, the de-regulated and inactive IPMS3 in *S. lycopersicum* and *S. pennellii* directs more carbon flow toward leucine and valine metabolism, respectively. Having the broad substrate specificity of downstream acyl-CoA-dependent acyltransferase (Schilmiller et al., 2015), increased availability of 3MOB and 3-isopropylmalate contributes to the formation of iC4 and iC5 acylsugars, respectively. Brassicaceae species including *Arabidopsis thaliana* also has a truncated IPMS homolog but with point mutations that alter substrate specificity to now function as methylthioalkylmalate synthase in the initial step of methionine-derived glucosinolate biosynthesis (de Kraker and Gershenzon, 2011). Unlike the latter example of recruitment of specialized metabolic enzymes from primary metabolism, as discussed in previous reviews (Weng, 2014; Moghe and Last, 2015), the study by Ning et al. (2015) revealed a role of altered branch chain amino acid biosynthesis in the acyl chain diversity of acylsugars in the *Solanum* genus.

Anthranilate synthase (AS) catalyzes the committed step of biosynthesis of an aromatic amino acid, L-tryptophan, and its enzyme activity is strictly regulated through feedback inhibition of one of the AS enzyme complex, ASα, by tryptophan (Romero et al., 1995; Li and Last, 1996). Two copies of *ASα* genes, *ASα1* and *ASα2*, were found in *Ruta graveolens* (the Rutaceae family) that uses anthranilate to produce unique specialized metabolites, acridone alkaloids (Bohlmann et al., 1995). While *ASα2* was constitutively expressed, *ASα1* was induced under elicitor treatment, which stimulates the accumulation of acridone alkaloids. Interestingly, the *ASα1* enzyme was much more resistant than *ASα2* to the tryptophan-mediated feedback inhibition, suggesting that the expression of the de-regulated ASα1 enzyme allowed elevated accumulation of the anthranilate precursor and hence efficient production of the downstream specialized metabolites, acridone alkaloids, in this unique plant lineage (Bohlmann et al., 1996; Figure 1). A naturally occurring feedback-insensitive ASα enzyme has also been identified in *Nicotiana tabacum* (the Solanaceae family, Song et al., 1998), but its *in planta* function is currently unknown. Further evolutionary analyses across the Rutaceae family can evaluate if the increased availability of anthranilate served as an enabling trait for *later* evolution of acridone alkaloid biosynthesis. Alternatively, the de-regulated ASα1 might have evolved *after* the emergence of the acridone alkaloid pathway as an associated trait and further elevated the alkaloid production.

L-Tyrosine is another aromatic amino acid required for protein synthesis but also used to produce diverse plant natural products, such as tocochromanols, benzylisoquinoline alkaloids, cyanogenic glycosides (e.g., dhurrin), and rosmarinic acids (Schenck and Maeda, 2018). Tyrosine is typically produced via arogenate dehydrogenase (TyrA_a_) that is localized within the plastids (Rippert et al., 2009; Wang et al., 2016) and strongly feedback inhibited by tyrosine (Figure 1; Connelly and Conn, 1986; Rippert and Matringe, 2002a,b). Recent studies, however, uncovered diversification of the tyrosine biosynthetic pathways in different plant lineages. In addition to the highly regulated plastidic TyrA_a_-mediated pathway, many legumes including *Glycine max* (soybean) and *Medicago truncatula* have an additional tyrosine biosynthetic pathway mediated by prephenate dehydrogenase (TyrA_p_) (Rubin and Jensen, 1979; Schenck et al., 2015), which is often found in microbes (Bonner and Jensen, 1987; Bonner et al., 2008; Schenck et al., 2017b). Notably, these legume TyrA_p_ enzymes are localized outside of the plastids and completely insensitive to feedback inhibition by tyrosine (Schenck et al., 2015, 2017a), suggesting that the alternative tyrosine pathway is physically separated from the canonical plastidic pathway and escaped feedback inhibition by tyrosine (Figure 1). While the metabolic and physiological functions of the alternative cytosolic TyrA_p_ pathway in legumes is largely unknown, some legumes accumulate very high levels of tyrosine and tyrosine-derived compounds (e.g., L-DOPA in *Mucuna pruriens*, Wichers et al., 1993; Lokvam et al., 2006). A recent study found that the expression of gene encoding the tyrosine-insensitive TyrA_p_ enzyme is elevated in *Inga* species that accumulate tyrosine and its derived secondary metabolites (e.g., tyrosine-gallates) at 5 to 20% of seedling dry weight (Coley et al., 2019). Thus, the presence of the feedback-insensitive TyrA_p_ enzyme in the legume family likely provided a unique pre-condition that enabled increased tyrosine biosynthetic activity and hyper-accumulation of tyrosine-derived compounds in this specific genus of legumes.

Betalains are red to yellow alkaloid pigments uniquely produced in the plant order Caryophyllales, which include *Beta vulgaris* (beet), spinach, quinoa, and cactus. Betalain pigments are derived from tyrosine and replaced more ubiquitous red to purple anthocyanin pigments derived from phenylalanine in many Caryophyllales species (Tanaka et al., 2008; Brockington et al., 2011; Polturak and Aharoni, 2018; Figure 1). Like Arabidopsis and unlike legumes, Caryophyllales species only have arogenate-specific TyrA_a_ enzymes; however, one TyrA_a_ isoform (TyrA_a_α) exhibits relaxed sensitivity to tyrosine inhibition (Lopez-Nieves et al., 2018; Figure 1). The presence of the de-regulated TyrA_a_α enzymes positively and negatively correlates with those of betalain and anthocyanin pigmentation, respectively, across Caryophyllales. Evolutionary analyses, by utilizing transcriptome data of over one hundred Caryophyllales species (Brockington et al., 2015), revealed that the de-regulated TyrA_a_α enzymes emerged *before* the evolution of the betalain biosynthetic pathway (Lopez-Nieves et al., 2018). Thus, the enhanced supply of the tyrosine precursor, due to relaxed regulation of the TyrA_a_ enzyme, likely acted as a metabolic enabler for the subsequent evolution of a novel downstream specialized metabolic pathway, betalain biosynthesis, in this specific plant order (Figure 1). Further evolutionary analyses of associated genes and enzymes involved in the betalain pathway and the competing phenylalanine and phenylpropanoid pathways will provide novel insight into how primary and specialized metabolism evolved coordinately in a macroevolutionary scale beyond the levels of species and genera.

## Ancient Diversification of IPP and Phenylalanine Biosynthetic Pathways in Plantae

In the ancient history of Plantae, alternative primary metabolic pathways evolved and likely contributed to later evolution of plant specialized metabolism and chemical diversity. Terpenoids and phenylpropanoids are the two major classes of plant natural products, which are synthesized from the primary metabolite precursors, isopentenyl pyrophosphate (IPP) and phenylalanine, respectively (McGarvey and Croteau, 1995; Gershenzon and Dudareva, 2007; Vogt, 2010; Tohge et al., 2013). Notably, plants possess two alternative pathways to synthesize IPP and phenylalanine.

In addition to sterols and quinones, plants use IPP to synthesize photosynthetic pigments (chlorophylls, carotenoids), plant hormones (brassinosteroids, abscisic acid, gibberellins), and a diverse array of terpenoid compounds (McGarvey and Croteau, 1995; Gershenzon and Dudareva, 2007; Tholl, 2015). Such a high demand of IPP for synthesis of diverse terpenoid compounds in plants is supported by the two alternative IPP biosynthetic pathways, the methylerythritol phosphate (MEP) and mevalonate (MVA) pathways, which take place in the plastidic and extra-plastidic subcellular compartments, respectively (Vranová et al., 2013; Rodríguez-Concepción and Boronat, 2015). The MEP pathway utilizes glyceraldehyde 3-phosphate derived from the pentose phosphate pathways in the plastids and hence can draw carbon flux directly from photosynthetic carbon fixation (Figure 1). While the MVA pathway appears to be an ancestral pathway that evolved in all three domains of life (i.e., eukaryotes, archaea, and most bacteria) or in their last universal ancestor (i.e., cenancestor) (Lombard and Moreira, 2011), the plastidic MEP pathway has mosaic evolutionary origins (Lange et al., 2000; Matsuzaki et al., 2008). A common ancestor of plastid bearing eukaryotes likely acquired MEP pathway enzymes from various bacterial ancestors (i.e., cyanobacteria, α-proteobacteria, Chlamydia) through horizontal gene transfers (Matsuzaki et al., 2008) and the MEP pathway was vertically transmitted to the descendants, the entire Plantae including algae and plants.

L-Phenylalanine is the primary metabolite precursor of phenylpropanoids and is synthesized via two alternative pathways in plants (Tzin and Galili, 2010; Maeda and Dudareva, 2012; Yoo et al., 2013; Qian et al., 2019). In many microbes, phenylalanine is synthesized via the phenylpyruvate intermediate, catalyzed by prephenate dehydratase (PDT) and phenylpyruvate aminotransferase (Figure 1) (Bentley, 1990). Although an analogous phenylpyruvate pathway also exists in the plant cytosol (Yoo et al., 2013; Qian et al., 2019), plants synthesize phenylalanine mainly in the plastids via the L-arogenate intermediate: prephenate is first transaminated by prephenate aminotransferase (PPA-AT) to arogenate (Graindorge et al., 2010; Dal Cin et al., 2011; Maeda et al., 2011), which is then converted to phenylalanine by arogenate dehydratase (ADT; Siehl and Conn, 1988; Cho et al., 2007; Maeda et al., 2010; Figure 1). Evolutionary analyses of the PPA-AT and ADT enzymes suggested that an ancestor of green algae and land plants appear to have acquired both of these two enzymes from an ancestor of Chlorobi/Bacteroidetes bacteria, likely through horizontal gene transfer (Dornfeld et al., 2014). Some cyanobacteria also have PPA-AT enzymes but with a distinct evolutionary origin from those of plants and Chlorobi/Bacteroidetes bacteria (Graindorge et al., 2014; Giustini et al., 2019). Thus, these dual primary metabolic pathways of isoprenoid and phenylalanine biosynthesis appear to have evolved in a common ancestor of Plantae. Although evolutionary analyses of such deep phylogenetic nodes are challenging, these dual precursor supply pathways potentially served as metabolic enablers for the evolutionary expansion of terpenoids and phenylpropanoids, the hallmarks of chemical diversity uniquely seen in the plant kingdom today.

## Diversification of Lipid Metabolism in Plants

Notable chemical diversity also exists in plant lipid metabolism (Badami and Patil, 1980; Ohlrogge et al., 2018), which makes the boundary of primary and specialized (secondary) metabolism difficult to define. Besides major acyl chains (e.g., oleic 18:1, linolenic 18:3) found in most plant lipids, some plants produce unusual fatty acids: For example, oils of castor (*Ricinus communis*, Euphorbiaceae family) and *Vernonia galamensis* (Asteraceae family) consist of primarily (80–90%) hydroxylated and epoxy fatty acids, respectively (Canvin, 1963; Ayorinde et al., 1990). Also, diverse acetylenic natural products having a carbon-carbon triple bond(s) or alkynyl functional group can be produced by modification of the fatty acid precursors (Minto and Blacklock, 2008; Negri, 2015). The production of these hydroxylated fatty acids and polyacetylenes are mediated by divergent fatty acid desaturases with altered product specificities and catalytic properties (van de Loo et al., 1995; Broun et al., 1998; Liu et al., 1998; Broadwater et al., 2002; Minto and Blacklock, 2008; Negri, 2015). Tremendous diversity of cuticular waxes has been also documented across the plant kingdom likely due to the presence of specialized acyl chain elongation and modifying enzymes (Jetter et al., 2007; Busta and Jetter, 2018).

Recent studies also revealed an intriguing alteration in the core lipid metabolic pathway, triacylglycerol (TAG) biosynthesis, in a specific plant lineage. The fruits of Bayberry (*Myrica pensylvanica*, Myricaceae family) accumulate abundant and unusual extracellular glycerolipids: TAG, diacylglycerol (DAG), and monoacylglycerol with completely saturated acyl chains at up to 30% of fruit dry weight (Harlow et al., 1965; Simpson and Ohlrogge, 2016). This unique surface wax attracts birds for seed dispersal and is used for making scented candles (Fordham, 1983). Fleshy fruits of oil palm, olive, and avocado also accumulate a large quantity of glycerolipids but intracellularly and by upregulating conventional fatty acid and TAG biosynthetic pathways (Bourgis et al., 2011; Kilaru et al., 2015). In contrast, a novel TAG biosynthetic pathway evolved in Bayberry through “re-purposing” genes and enzymes involved in cutin biosynthesis by altering their gene expression (Simpson and Ohlrogge, 2016; Simpson et al., 2016). These alterations include elevated expression of genes encoding the G subfamily of ABC (ABCG) transporters and lipid transporter proteins likely required for lipid transport across cell membranes and walls, respectively, which will allow extracellular formation of TAG (Simpson et al., 2016). It will be interesting to examine how such reprograming of existing lipid metabolic pathways occur in a step-wise manner during evolution, which will provide useful information for engineering other plants to produce and secrete abundant extracellular glycerolipids.

## Summary and Perspective

Although not as frequent as those of specialized metabolism, accumulating evidence indicates that pathways and enzymes of primary metabolism can be diversified during the plant evolution. Such relatively rare alterations in primary metabolism likely contributed to major evolutionary innovations in the plant kingdom, including the evolution of downstream specialized metabolic pathways and hence plant chemical diversity. Some alterations in primary metabolism appear to have acted as enabling traits for the evolution of novel specialized metabolism, at least in the case of de-regulated tyrosine biosynthesis in Caryophyllales that preceded the emergence of betalain pigmentation (Lopez-Nieves et al., 2018). In other instances, primary metabolic alterations likely co-evolved with and support efficient operation of specialized metabolic pathways. It remains to be examined how prevalent the phenomenon is beyond the pathways and plant lineages that have been examined so far and what impacts such primary metabolic diversification had on overall metabolism, physiology, and environmental adaption of diverse plant species. Another intriguing question is how seemingly maladaptive alterations in highly conserved and constrained primary metabolism were maintained in certain plant lineages, especially until the emergence of a new downstream pathway which might have eventually provided adaptive advantage. What are the environmental, anatomical, and genetic enablers underlying primary metabolic diversification? In the case of tomato feedback-insensitive IPMS and legume TyrA_p_ enzymes, their specific expression in the apical trichome cells (Ning et al., 2015) and extra-plastidic subcellular compartment (Schenck et al., 2015) likely allow minimal disturbance to *de novo* biosynthesis of branch chain and aromatic amino acids, respectively. Further addressing these questions will lead to broader understanding of the evolution of plant metabolism at a macroevolutionary scale. The acquired knowledge of primary metabolic diversification and its underlying genetic and biochemical basis will also allow us to redesign plant metabolism in a holistic manner from primary to specialized metabolism.

## Author Contributions

HM wrote the manuscript.

## Conflict of Interest Statement

The author declares that the research was conducted in the absence of any commercial or financial relationships that could be construed as a potential conflict of interest.
